# Enhancement of Cellulose Degradation by Cattle Saliva

**DOI:** 10.1371/journal.pone.0138902

**Published:** 2015-09-24

**Authors:** Yasutaka Seki, Yukiko Kikuchi, Yoshihiro Kimura, Ryo Yoshimoto, Masatoshi Takahashi, Kenichi Aburai, Yoshihiro Kanai, Tatsushi Ruike, Kazuki Iwabata, Fumio Sugawara, Hideki Sakai, Masahiko Abe, Kengo Sakaguchi

**Affiliations:** 1 Research Institute for Science and Technology, Tokyo University of Science, Noda, Chiba, Japan; 2 Department of Industrial Chemistry, Tokyo University of Science, Noda, Chiba, Japan; 3 Department of Applied Biological Science, Tokyo University of Science, Noda, Chiba, Japan; Korea University, REPUBLIC OF KOREA

## Abstract

Saccharification of cellulose is a promising technique for producing alternative source of energy. However, the efficiency of conversion of cellulose into soluble sugar using any currently available methodology is too low for industrial application. Many additives, such as surfactants, have been shown to enhance the efficiency of cellulose-to-sugar conversion. In this study, we have examined first whether cattle saliva, as an additive, would enhance the cellulase-catalyzed hydrolysis of cellulose, and subsequently elucidated the mechanism by which cattle saliva enhanced this conversion. Although cattle saliva, by itself, did not degrade cellulose, it enhanced the cellulase-catalyzed degradation of cellulose. Thus, the amount of reducing sugar produced increased approximately 2.9-fold by the addition of cattle saliva. We also found that non-enzymatic proteins, which were present in cattle saliva, were responsible for causing the enhancement effect. Third, the mechanism of cattle saliva mediated enhancement of cellulase activity was probably similar to that of the canonical surfactants. Cattle saliva is available in large amounts easily and cheaply, and it can be used without further purification. Thus, cattle saliva could be a promising additive for efficient saccharification of cellulose on an industrial scale.

## Introduction

In recent years, we have been facing shortages in fossil fuels that are widely used as energy sources. Because of this shortage in fossil fuels, biomass energy has been receiving a lot of attention as an alternative energy source. The most widely used method for obtaining biomass energy is the conversion of cellulose into bioethanol as a fuel [1, 2]. Cellulose is the preferred raw material for producing new sources of energy because it is the most abundantly available organic polymer in the world. However, relatively low conversion efficiency of cellulose to soluble sugars is a bottleneck for its industrial application. Generally, enzymatic hydrolysis by cellulase is one of the most commonly used methods for converting cellulose into soluble sugar. Cellulose is a rigid molecule because of its crystalline structure, as a consequence of which cellulose becomes inaccessible to the hydrolyzing enzyme cellulase [3].

To circumvent this problem, many researchers have attempted to efficiently convert cellulose into soluble sugars by using additives. As a result, several authors have reported that enzyme catalyzed hydrolysis of cellulose could be enhanced by the addition of a number of surfactants. For example, Castanon and Wilke [4] showed that the addition of surfactant Tween 80 enhanced the rate of enzymatic hydrolysis of newspaper cellulose by 14%. In another study, Ooshima et al [5] showed that the surfactant-mediated enhancement of hydrolysis was higher for the crystalline cellulose than for the amorphous cellulose. Moreover, several cationic surfactants, but not anionic surfactants, enhanced the hydrolysis process [5]. There were also several other studies demonstrating the ability of surfactants in enhancing the rate of hydrolysis [6–8]. One study showed that the non-ionic surfactants were more effective at low cellulase concentration [9]. Addition of certain non-catalytic proteins, such as bovine serum albumin (BSA), was also shown to have an effect similar to the effect of addition of non-ionic surfactants. For example, the addition of 1.7% BSA to steam-pretreated spruce enhanced the hydrolysis process in a similar manner as was observed with the addition of Tween 20. But Tween 20 did not have any enhancement effect when added to a reaction mixture that contained BSA. A number of mechanisms have been proposed to explain how surfactants enhanced the hydrolysis of lignocelluloses. First, surfactants have been proposed to enhance the hydrolysis of lignocelluloses by acting on lignin, which inhibits the activity of cellulase by irreversibly binding to it [10]. BSA was also shown to have a similar effect [11]. Second, surfactants have been proposed to increase the stability of cellulase by reducing its denaturation during hydrolysis, and thereby enhancing the hydrolysis process [12, 13]. Third, surfactants have been proposed to have an effect on cellulase-substrate interaction, by preventing the dissociation of cellulase from the bound substrate [12].

In the present study, we searched for a new additive based on a known biological fact: herbivorous animals–such as cows, horses and goats–obtain energy needed for their survival from grass and other plants. We, therefore, reasoned that the herbivorous animals must possess an effective mechanism for converting cellulose, the major constituent of grass and plants, into a consumable source of energy, namely sugar. For example, cows mainly eat grass, and rumination enables them to chew grass more completely, which makes grass digestible. During the mastication process, cattle saliva is believed to play an assisting role in making the food digestible, as mastication physically destroys the rigid structure of cellulose. Thus, we thought that cattle saliva, as an additive, could also assist in enzymatic digestion of cellulose by interacting with it. In the present study, we have examined the properties of cattle saliva in degrading cellulose, and subsequently elucidated the mechanism by which cattle saliva enhances the enzyme catalyzed hydrolysis of cellulose.

## Materials and Methods

### Materials

Microcrystalline cellulose was purchased from Merck Co., Ltd. (Darmstadt, Germany) and used as the cellulose substrate. Timothy hay was used as the real biomass substrate. This contains 30% of cellulose and 10% of protein [14]. Cellulase, purified from *Trichoderma viride*, was purchased from Sigma-Aldrich Co., Ltd. (St Louis, MO, USA). Cellulase was solubilized in 50 mM sodium acetate buffer (pH 4.0) before use. Cattle saliva was obtained from female Holstein cows of Iwai ranch with the owner’s permission. The cattle saliva, secreted from the mouths of cows, was collected prior to feeding them, without causing any suffering and without invading their welfare. After collection, cattle saliva was centrifuged (10,000 x *g*, 4°C, 10 min) to remove any particulate material and the resulting supernatant was subsequently used to perform experiments.

### Determination of Reducing Sugar Concentration

The basic experimental protocol used for determining the reducing sugar concentration was as follows. One hundred sixty microliters cellulose suspension (1 wt%) was mixed with 20 μL cellulase solution (100 μg/mL) and 20 μL cattle saliva. Thus, the mixture (200 μL) contained 0.8 wt% cellulose, 10 μg/mL cellulase and 10% cattle saliva. The mixture was incubated at 50°C for 24 h. After incubation, the mixture centrifuged (10,000 x g, 4°C, 10 min), and the resulting supernatant was mixed with Somogyi-Nelson reagent. The concentration of reducing sugar in the mixture was determined by measuring the absorption at 595 nm using a SpectraMax 190 Microplate Reader (Molecular Devices LLC.; Sunnyvale, CA, USA). The cellulose conversion rate to reducing sugar for pure cellulose was calculated by the following equation:

$$Rp=\frac{200\;(\mu L)\times Cs}{200000\;(\mu g)\times 0.008}\times 100$$

*Rp* means cellulose conversion rate to reducing sugar for pure cellulose (percent) and *Cs* means sample reducing sugar concentration (microgram per microliter). Timothy hay (0.2 g) was suspended in sodium acetate buffer (20 mL). The suspension was pulverized at 750 rpm for 2 hours by using a planetary ball mill, P-7 (Frisch Ltd.; Haan, Germany). The ball-milled suspension was used as the real biomass substrate. The basic experimental protocol was as follow. Timothy hay suspension (1 wt%: 80 μL) was mixed with cellulase solution (500 μg/mL: 10 μL) and cattle saliva (100%: 10 μL). Thus, the mixture (100 μL) contained 0.8 wt% timothy hay, 50 μg/mL cellulase and 10% cattle saliva. The mixture was incubated at 50°C for 24 h. In this assay, the degree of cellulose degradation was determined by measuring the amount of glucose released from cellulose. Glucose concentration was measured by the Mutarotase-God method [15] using a LabAssay Glucose kit (Wako Pure Chemical Industries, Ltd.; Osaka, Japan). The cellulose conversion rate to glucose for the real biomass substrate was calculated by the following equation:

$$Rr=\frac{100\;(\mu L)\times (Cs-Ci)}{100000\;(\mu g)\times 0.008\times 0.3}\times 100$$

*Rr* means cellulose conversion rate to glucose for the real biomass substrate (percent), *Cs* means sample glucose concentration (microgram per microliter) and *Ci* means initial glucose concentration (microgram per microliter).

### Removal of Protein

Cattle saliva was treated with methanol or acetone to remove protein. One milliliter cattle saliva was mixed with 3 mL methanol or acetone. The acetone mixture was incubated at -30°C for 2 hours. The two mixtures were centrifuged (10,000 x *g*, 4°C, 5 min) and the supernatants were collected. The supernatants were evaporated and dried by rotary evaporator, RE300 (Yamato Scientific Co., Ltd.: Tokyo, Japan) and well dissolved in 1 mL sodium acetate buffer.

### Fourier Transform Infrared (FT-IR) Spectroscopy and X-ray Diffraction (XRD) Spectroscopy

Changes in the structural properties of cellulose were measured using FT-IR and X-ray diffraction spectroscopies. Infrared spectra measurements were carried out using a JASCO FT-IR 6100 spectrometer (JASCO Inc.; Tokyo, Japan) fitted with a KBr pellet. X-Ray diffraction measurements were carried out using a PHILIPS X’Pert Pro difractometer (Philips; Amsterdam, Netherlands) and CuKα radiation (45 kV acceleration voltage and 40 mA tube current). The observed diffraction angles ranged from 10 to 30 degrees.

### Gel-filtration Chromatography

Proteins in the cattle saliva were fractionated using a GE AKTAprime liquid chromatography system (GE Healthcare; Pittsburgh, PA, USA) equipped with a HiLoad 16/600 Superdex 200 prep grade column (GE Healthcare). The mobile phase used was an aqueous solution containing 150 mM NaCl. The injection volume was 4 mL and the flow rate was 1.0 mL min^-1^. Twenty-four fractions (each 5 mL) were collected, the collected fractions were then analyzed by SDS-PAGE and based on the obtained results, these fractions were subsequently divided into five sample groups, A through E. Fractions belonging to the same sample group were combined, the final volume of each of which was concentrated to 4 mL by ultrafiltration using an Amicon Ultra-15 3K filter (Merck Millipore Co., Ltd.; Darmstadt, Germany). A mixed sample (Mix) was prepared by combining 0.4 mL of each sample. Protein concentration in each sample was measured using the Bradford protein assay method. Each sample was diluted with an appropriate amount of 150 mM NaCl to obtain a final protein concentration of 40 μg/mL. An aliquot of each diluted sample was used as an additive in the enzyme-catalyzed cellulose degradation assay. Enzymatic activity of cellulase was determined by measuring the amount of glucose produced in each reaction mixture by using the Mutarotase-God method as described above.

### Addition Order Assay

To determine the effect of addition order, we prepared five different addition order mixtures as follows for comparing results. In the beginning, we prepared these mixtures by adding cellulose and cattle saliva to the first tube, cellulase and cattle saliva to the second tube, nothing to the third tube, cellulose and cellulase to the fourth tube, and cellulose, cellulase and cattle saliva to the fifth tube. These mixtures were then incubated at 50°C for 1 h. After the incubation, we added cellulase to the first tube (Added with cellulase), cellulose to the second tube (Added with cellulose), cellulase, cellulose and cattle saliva to the third tube (Simultaneous), cattle saliva to the fourth tube (Added with saliva), and nothing to the fifth tube (Simultaneous, 25 h). All of them were then incubated at 50°C for an additional period of 24 h. Enzymatic activity of cellulase was determined by measuring the amount of glucose produced in each reaction mixture by using the Mutarotase-God method as described above.

### Assay for Measuring Adsorption of Cattle Saliva Proteins to Cellulose

Adsorption of cattle saliva proteins to cellulose was analyzed as follows using SDS-PAGE and Bradford protein assay. Briefly, 160 μL cellulose suspension (containing 1.6 mg cellulose) was dispensed into an experimental tube. After cellulose was settled at the bottom of the tube, 144 μL of clear supernatant was carefully removed and discarded. Then 20 μL cattle saliva was added to the tube. The mixture was incubated at 50°C for 1 h, following which 20 μL supernatant was collected as a ‘Supernatant’ fraction. In order to wash out the unabsorbed proteins, the cellulose pellet was washed three-times with 50 mM sodium acetate buffer (20 μL each time), and supernatant (20 μL each) from each wash (called as ‘Wash 1’, ‘Wash 2’ and ‘Wash 3’ fractions) was collected for further analysis. Following the third wash, 20 μL of 50 mM sodium acetate buffer was added to the cellulose pellet. Finally, 5 μL SDS-PAGE loading buffer (containing 2.5% SDS, 50% glycerol and 375 mM Tris-HCl) was added to all the collected fractions and to the cellulose pellet. All mixtures were heated at 96°C for 1 h to degenerate proteins. The supernatant obtained from the cellulose pellet containing mixture was collected as the ‘Elute’ fraction. Aliquots from each fraction were taken out for the SDS-PAGE analysis and Bradford protein assay.

### Competition between Cattle Saliva and Canonical Additive

An assay was performed to determine competition between cattle saliva and Tween 20, as Tween 20 has been used as a typical canonical additive for enhancing cellulose degradation. Briefly, 160 μL of 1% (wt%) cellulose suspension was mixed with 20 μL of 100 μg/mL cellulase solution, 15.5 μL of cattle saliva and 4.5 μL of 10% Tween 20. Thus, 200 μL of the assay mixture contained 1.6 mg cellulose, 2.0 μg cellulase (10 μg/mL final), 7.75% cattle saliva and 11.25 μg Tween 20 (2.5 mg/mL final). The assay mixture was incubated at 50°C for 24 h and the amount of reducing sugar produced in the supernatant was quantified by Somogyi-Nelson method (see above).

## Results & Discussion

### Properties of Cattle Sativa

**Fig 1:**
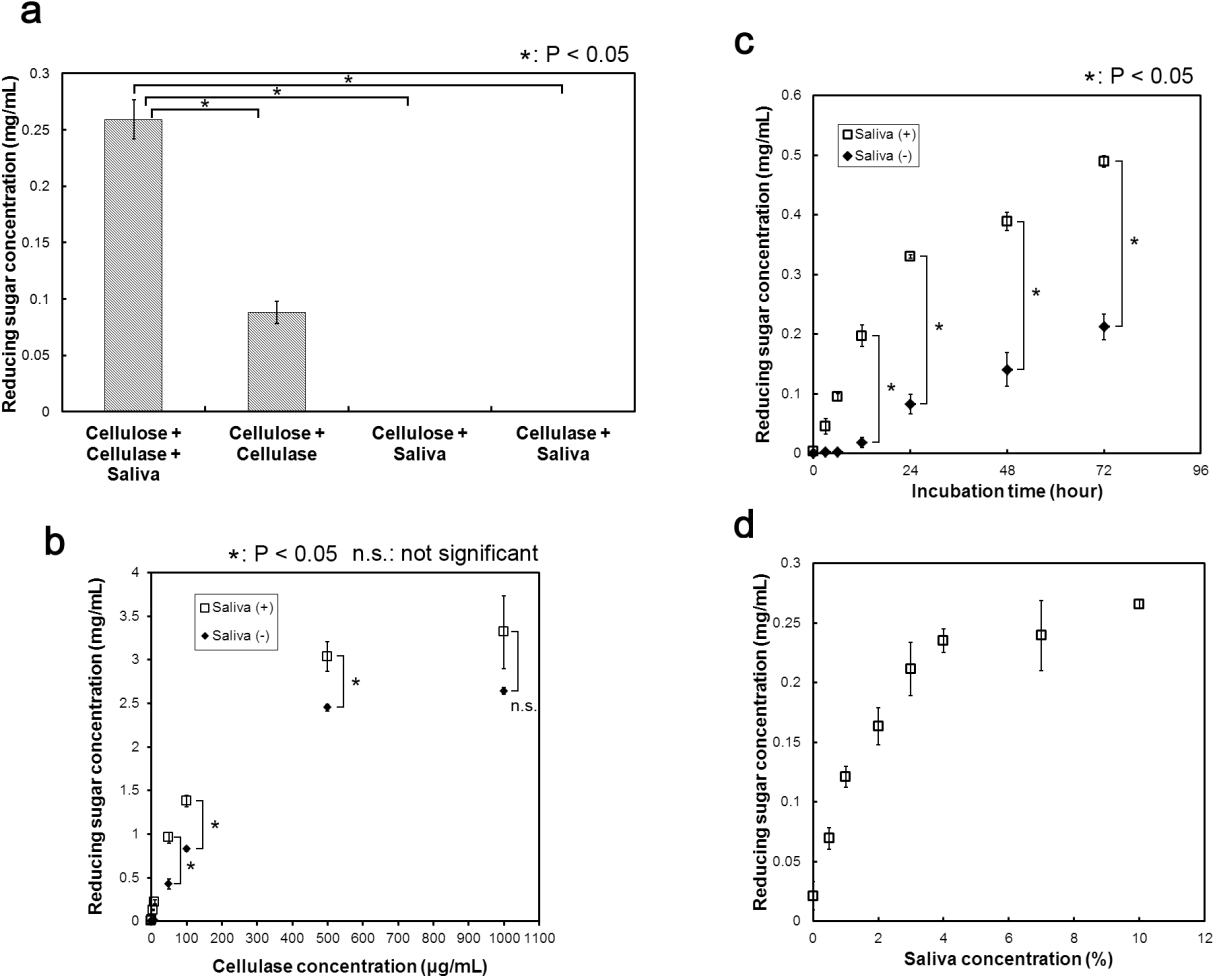
Effects of cattle saliva on cellulose degradation. (a) Enhancement effect of cattle saliva. Effect of cattle saliva addition on the production of reducing sugar from micro-crystalline cellulose. Reaction mixtures containing 10 μg/mL cellulase and 0.8% (wt%) cellulose were incubated in the presence or absence of 10% cattle saliva at 50°C for 24 h. Effects of (b) cellulase concentration, (c) incubation time and (d) cattle saliva concentration on reducing sugar production. In (b), concentrations of cellulase used were 0, 1, 5, 10, 50, 100, 500 and 1000 μg/mL, while the concentration of cellulose was kept same as in (a) above and the reaction mixtures were incubated at 50°C for 24 h. In **(c),** different incubation times were used (0, 1, 3, 6, 12, 24, 48 and 72 h) while keeping the composition of the reaction mixture same as in (a) above. In (d), different concentrations of cattle saliva were used here: 0, 0.5, 1, 2, 3, 4, 7 and 10%; concentrations of cellulase and cellulose and reaction conditions were same as in (a) above. All experiments were performed in triplicate and results are expressed as average means. Error bars indicate ± standard deviations. Values labeled with asterisk are statistically different as established by Student's t-test (P < 0.05).

First, we examined the effect of cattle saliva on cellulose hydrolysis. Fig 1A shows the results of adding cattle saliva to a mixture containing microcrystalline cellulose and cellulase (Cellulose + Cellulase + Saliva). We also confirmed the effect of the combinations of ‘Cellulose + Cellulase’, ‘Cellulase + Saliva’ and ‘Cellulase + Saliva’ on cellulose degradation, as controls. The concentrations of reducing sugar (and the conversion rates to reducing sugar) found in the ‘Cellulose + Cellulase + Saliva’ mixture, ‘Cellulose + Cellulase’ mixture, ‘Cellulose + Saliva’ mixture and ‘Cellulase + Saliva’ mixture were 0.259 mg/mL (3.24%), 0.088 mg/mL (1.10%), not detected and not detected, respectively. Thus, the amount of reducing sugar produced from cellulose increased approximately 2.9-fold by the addition of cattle saliva. Together, these results suggested that cattle saliva does not degrade cellulose by itself, but enhances cellulase-catalyzed hydrolysis of cellulose. We obtained virtually same results using saliva from other cattle (data not shown), thus suggesting that the observed enhancing effect of cattle saliva was not individual specific.

We next characterized the effect of cattle saliva on cellulase activity in more detail. First, we examined the effect of varying cellulase concentration on the cellulase activity (Fig 1B). At 50 μg/mL cellulase loading, the reducing sugar concentrations (and cellulose conversion rates to reducing sugar) produced with and without cattle saliva were 0.959 mg/mL (11.99%) and 0.428 mg/mL (5.35%). At 500 μg/mL cellulase loading, the reducing sugar concentrations (and cellulose conversion rates to reducing sugar) produced with and without cattle saliva were 3.037 mg/mL (37.96%) and 2.452 mg/mL (30.65%). At 1,000 μg/mL cellulase loading, the reducing sugar concentrations (and cellulose conversion rates to reducing sugar) produced with and without cattle saliva were 3.317 mg/mL (41.47%) and 2.641 mg/mL (33.01%). At a low cellulase loading (less than 500 μg/mL), the amount of reducing sugar produced in both Saliva (+) and Saliva (-) reaction mixtures increased almost linearly with the corresponding increase in cellulase concentration. However, at a high cellulase loading (more than 500 μg/mL), the increased rate of sugar production slowed down and eventually reached saturation. These results also showed that the cattle saliva was able to enhance the degradation of cellulose at each cellulase concentration. Furthermore, the reducing sugar concentration became saturated at a higher cellulase concentration for the Saliva (+) reaction mixture than that for the Saliva (-) reaction mixture. These results suggested that the observed increase in cellulase activity was not limited by the amount of cellulase used in the reaction mixture, but due to the fact that the activity of celluase was enhanced by the added cattle saliva. Second, we compared the effect of incubation time on cellulose degradation (Fig 1C). In the mixture incubated for 12 hours, the reducing sugar concentrations (and cellulose conversion rates to reducing sugar) produced with and without cattle saliva were 0.197 mg/mL (2.46%) and 0.018 mg/mL (0.23%). In the mixture incubated for 24 hours, the reducing sugar concentrations (and cellulose conversion rates to reducing sugar) produced with and without cattle saliva were 0.330 mg/mL (4.13%) and 0.083 mg/mL (1.03%). In the mixture incubated for 48 hours, the reducing sugar concentrations (and cellulose conversion rates to reducing sugar) produced with and without cattle saliva were 0.389 mg/mL (4.86%) and 0.141 mg/mL (1.76%). In the mixture incubated for 72 hours, the reducing sugar concentrations (and cellulose conversion rates to reducing sugar) produced with and without cattle saliva were 0.489 mg/mL (6.11%) and 0.212 mg/mL (2.65%). As can be seen, the production of reducing sugar was increased with the corresponding increase in incubation time. When the incubation time was less than 24 h, the rate of sugar production in the Saliva (+) mixture was higher than that in the Saliva (-) mixture. However, when incubation time was more than 24 h, the rate of sugar production in the Saliva (+) mixture was almost same as that in the Saliva (-) mixture. These results suggested that cattle saliva accelerated the degradation reaction of cellulose from the very beginning. Third, we measured the amount of reducing sugar produced at various cattle saliva concentrations (Fig 1D). Using 0, 0.5, 1, 2, 3 and 4% of cattle saliva as an additive, we obtained 0.021, 0.069, 0.121, 0.163, 0.211 and 0.235 mg/mL, respectively, of reducing sugar. The production of reducing sugar increased almost linearly for up to 4% of cattle saliva. However, at concentrations of more than 4% of cattle saliva, the amount of sugar (and cellulose conversion rate to reducing sugar) produced seem to be leveling off around 0.25 mg/mL (3.13%). There result suggested that cattle saliva can effectively enhance the degradation of cellulose only up to a certain extent, beyond which the enhancement effect of cattle saliva becomes molecularly saturated.

**Fig 2:**
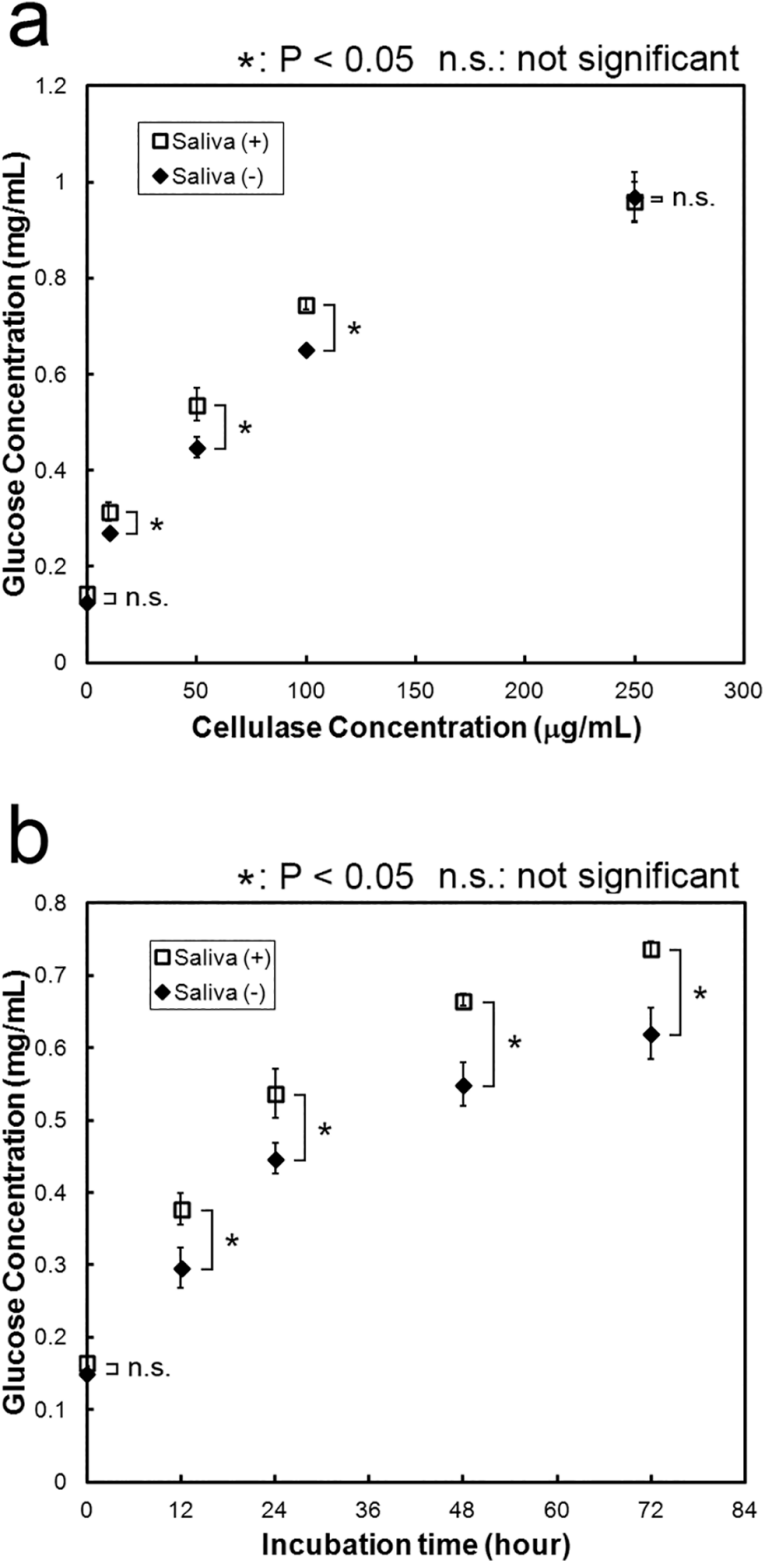
Properties of cattle saliva on real biomass degradation. Effects of (a) cellulase concentration and (b) incubation time on cellulose conversion. (a) Cellulase concentrations were 0, 10, 50, 100 and 250 μg/mL, while concentration of cattle saliva was constantly 10%. The reaction mixtures were incubated at 50°C for 24 h. (b) Different incubation times were tested (0, 12, 24, 48 and 72 h), while concentrations of cellulase and cattle saliva were constantly 50 μg/mL and 10%, respectively. The reaction mixtures were incubated at 50°C. All experiments were performed in triplicate and average mean values were plotted. Error bars indicate ± standard deviations. Values labeled with asterisk are statistically different as established by Student's t-test (P < 0.05).

We also characterized enhancing effect of cattle saliva for the real biomass substrate (Fig 2). First, approximately 0.15 mg/mL of glucose was detected in the non-reacted mixture. Timothy hey originally contained slight glucose. At 50 μg/mL celluase loading, the glucose concentrations (and cellulose conversion rates to glucose) in the mixtures with and without cattle saliva were 0.537 mg/mL (16.34%) and 0.448 mg/mL (13.38%). At 250 μg/mL celluase loading, the glucose concentrations (and cellulose conversion rates to glucose) in the mixtures with and without cattle saliva were 0.960 mg/mL (33.97%) and 0.968 mg/mL (35.07%). The enhancement effect occurred at a low enzyme loading (less than 100 μg/mL), paralleled with the result in pure cellulose experiment, whereas the enhancement effect did not occurred at a high enzyme loading (250 μg/mL) (Fig 2A). The maximum enhancement effect was 1.2-fold at 50 μg/mL celluase loading. In addition, the cellulose conversion rates produced with and without cattle saliva were higher for the real biomass substrate (16.34% and 13.38%) than that for pure cellulose (11.99% and 5.35%) at 50 μg/mL celluase loading (Fig 1B). We next measured the effect of reaction time for cellulose conversion at 50 μg/mL celluase loading (Fig 2B). The amount of glucose produced higher in the mixture with cattle saliva than in the mixture without cattle saliva. After incubation for 12 hours, the glucose concentrations (and cellulose conversion rates to glucose) in the mixtures with and without cattle saliva were 0.377 mg/mL (8.85%) and 0.296 mg/mL (6.06%). After incubation for 72 hours, the glucose concentrations (and cellulose conversion rates to glucose) in the mixtures with and without cattle saliva were respectively 0.737 mg/mL (23.84%) and 0.620 mg/mL (19.54%). The enhancement effect was approximately 1.2-fold when the incubation times were from 12 to 72 hours. When the incubation time was 12 hours, the rate of sugar production in the Saliva (+) mixture was higher than that in the Saliva (-) mixture. However, when incubation time was more than 12 hours, the rate of sugar production in the Saliva (+) mixture was almost same as that in the Saliva (-) mixture. Initial acceleration for the real biomass substrate degradation occurred until at least 12 hours in addition of cattle saliva. Cattle saliva also accelerated the degradation reaction of pure cellulose until 24 hours (Fig 1C). The changes of glucose production depended on the incubation time between the real biomass substrate and pure cellulose were similar. These results also showed that the enhancement rate for the real biomass substrate (1.2-fold) was lower than for pure cellulose (2.9-fold) (Figs 1A and 2A). The real biomass substrate, such as timothy hay, contains various types of organic substances and minerals, including protein, pectin, lignin, fat, calcium and phosphorus. The enhancement effect of cattle saliva may compete with some kind of substance in real biomass. Indeed, the cellulose conversion produced without cattle saliva for the real biomass (13.38%) was much higher than that for pure cellulose (5.35%). We identified the substance in cattle saliva responsible for enhancement effect in the next section in order to delineate the mechanism of enhancement effect, including the low enhancement effect for the real biomass substrate.

### Identification of the Substance or Factor in Cattle Saliva Responsible for Enhancing Cellulose Degradation

**Fig 3:**
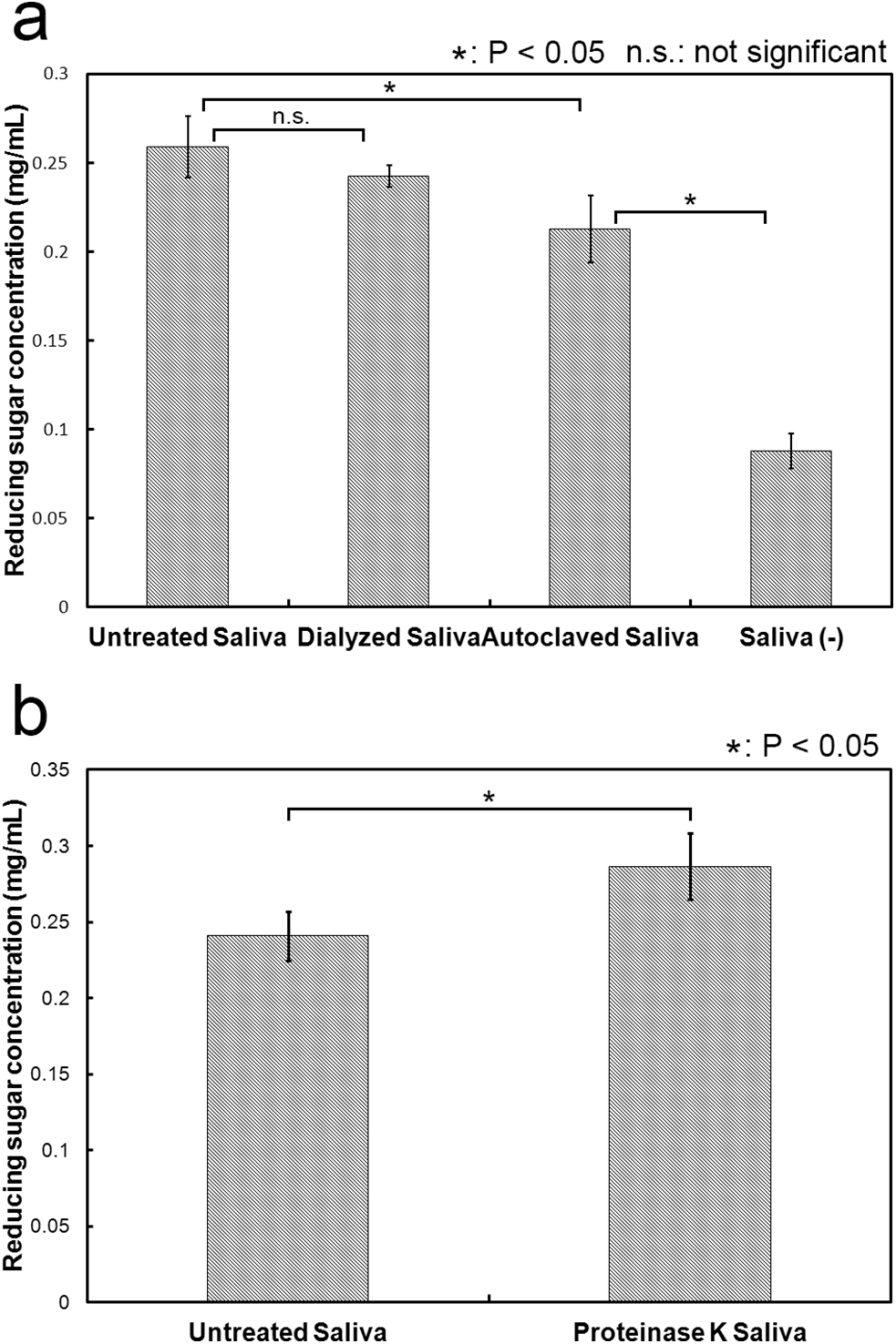
Effect of various treatments on the enhancement effect of cattle saliva. (a) Denatured and dialyzed cattle saliva. Denaturation of cattle saliva: Cattle saliva was autoclaved for 13 minites at 121°C to denature proteins. After that, the saliva was centrifuged at 20,400 x *g* for 10 min. The supernatant (called ‘Autoclaved saliva’) was collected and subsequently used in experiments. Dialysis of cattle saliva: Cattle saliva was dialyzed against distilled water for 72 h at room temperture. The water was exchanged every other day. (b) Proteinase K treatment. Twenty microliters cattle saliva was mixed with 20 μL proteinase K (20 mg/mL) and the mixture was incubated at 50°C for 12 h. After the incubation, the mixture was incubated at 96°C for 10 min to denature proteinase K. This mixture was called ‘Proteinase K Saliva’ and used in the cellulose degradation assay. The concentration of cattle saliva in the reaction mixture was 5%. All experiments were performed in triplicate and average mean values were plotted. Error bars indicate ± standard deviations. Values labeled with asterisk are statistically different as established by Student's t-test (P < 0.05).

We next attempted to identify the substance in the cattle saliva that was responsible for enhancing the degradation of cellulose. Cattle saliva contained many different types of minerals and organic substances, including sialic acid, mucin, lactoferrin, IGF-1, sodium bicarbonate, phosphoric salt and metal ions. In order to determine whether the substance is a small or a polymer molecule, we first dialyzed the cattle saliva and then used the dialyzed cattle saliva in the cellulose degradation assay. As shown in Fig 3A, we observed no significant difference between the amounts of reducing sugar produced using dialyzed cattle saliva and un-dialyzed cattle saliva. As the pore size of the dialysis membrane is 25–50 Å, only small molecules of sizes less than 14 kDa, such as sialic acid, IGF-1, sodium bicarbonate, phosphoric salt and metal ions, would diffuse across this membrane during dialysis. As a result, these small molecules are expected to be absent in the dialyzed cattle saliva. Thus, these small molecules in cattle saliva were not responsible for enhancing the enzymatic activity of cellulase. Incidentally, most of the polymer molecules known to be present in the cattle saliva are proteins and glycoproteins.

Next, we examined whether the enhancement effect was caused by the enzymatic reaction of a protein present in the cattle saliva. To test this possibility, we used autoclaved and proteinase K-treated cattle saliva in the degradation assay. As shown in Fig 3A, the amounts of sugar produced with the not-autoclaved (Untreated) cattle saliva, autoclaved cattle saliva and without cattle saliva were respectively 0.259 mg/mL 0.213 mg/mL and 0.088 mg/mL. The produced sugar rate using autoclaved cattle saliva was 82% based on using untreated cattle saliva but did not much decrease compared to the mixture without saliva (34%). Meanwhile, the amounts of sugar produced using the untreated and proteinase K-treated were 0.241 mg/mL and 0.286 mg/mL, respectively (Fig 3B). The amount of sugar produced using the proteinase K-treated was approximately 1.2-fold higher than using the untreated cattle saliva. Proteins in cattle saliva were digested and fragmented to small size of protein or peptide by proteinase K. This result suggested that the small increase of enhancement effect was caused by the addition of proteinase K as non-enzymatic protein behavior or the fragmentation of protein. These results suggested that the enhancement effect was not caused by the enzymatic reaction of one or more of the proteins present in the cattle saliva. The results of dialysis, autoclave and proteinase K treatment experiments indicated that the substance responsible for enhancement effect seemed to be non-enzymatic protein.

**Fig 4:**
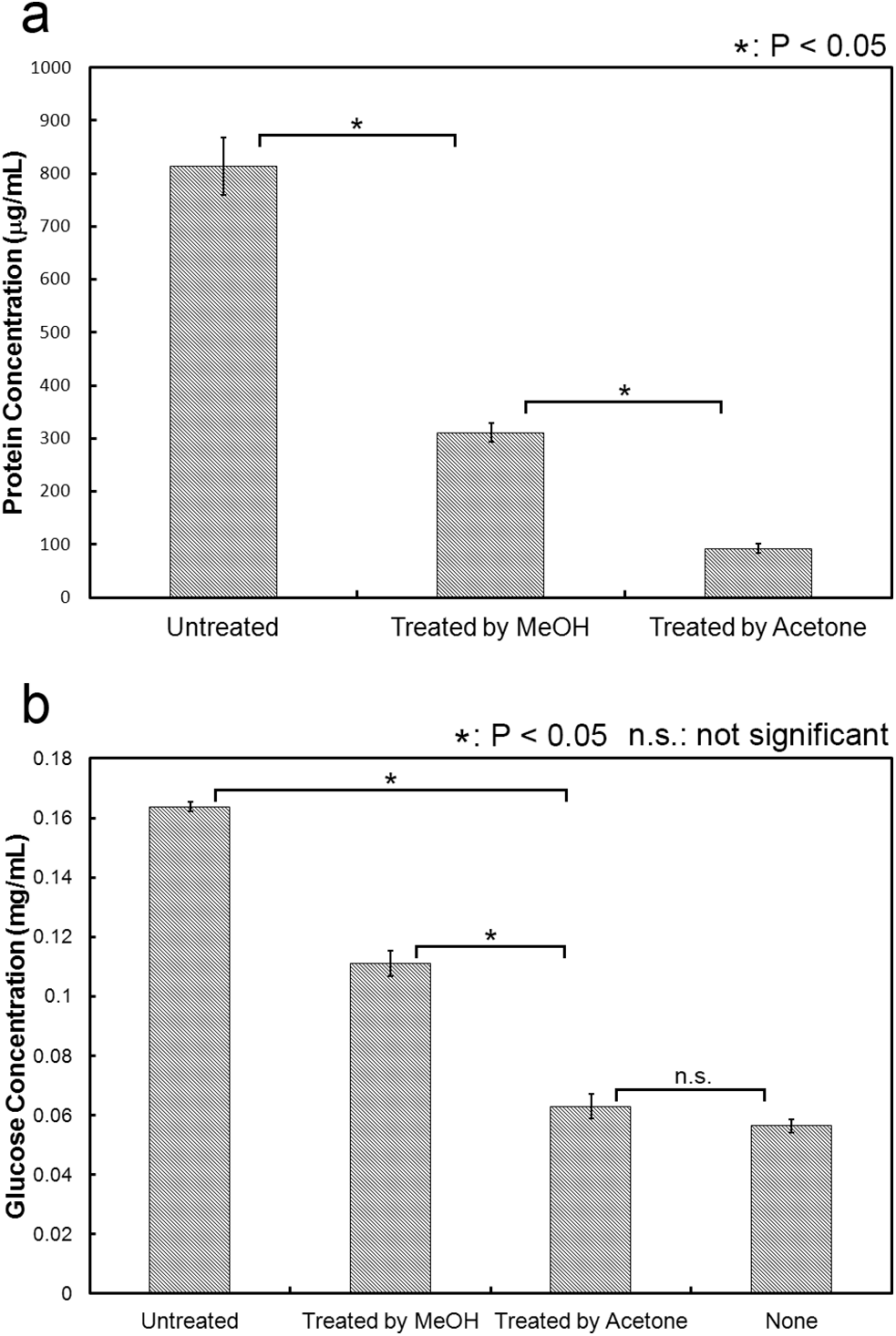
Removal of protein in cattle saliva. (a) Protein concentration in cattle saliva treated with methanol or acetone. Protein concentration was measured using Bradford protein assay. (b) Enhancement effect of cattle saliva treated with methanol or acetone. Cattle saliva treated with methanol or acetone used in the cellulose degradation assay. The reaction condition follows the basic experimental protocol. All experiments were performed in triplicate and average mean values were plotted. Error bars indicate ± standard deviations. Values labeled with asterisk are statistically different as established by Student's t-test (P < 0.05).

In order to remove proteins in cattle saliva, we treated cattle saliva with methanol and acetone. Methanol and acetone often denature and insolubilize protein and usually use for removal of protein. Meanwhile, various kinds of organic substances, except for protein, can be dissolved in methanol and acetone. We measured the protein concentration in cattle saliva treated with methanol or acetone by Bradford protein assay (Fig 4A). Proteins in cattle saliva treated with methanol contained more than treated with acetone. The rate of dissolved protein in untreated cattle saliva, cattle saliva treated with methanol and acetone were 100%, 38% and 11%, respectively. Based on the result of protein concentration in cattle saliva treated with methanol and acetone, we tested cattle saliva treated with methanol and acetone in the cellulose degradation assay (Fig 4B). The amount of glucose produced lower in cattle saliva treated with methanol or acetone than in untreated cattle saliva. The enhancement effect decreased corresponding decrease of the protein amount in cattle saliva. These results also suggested that the substance responsible for enhancement effect probably non-enzymatic protein.

**Fig 5:**
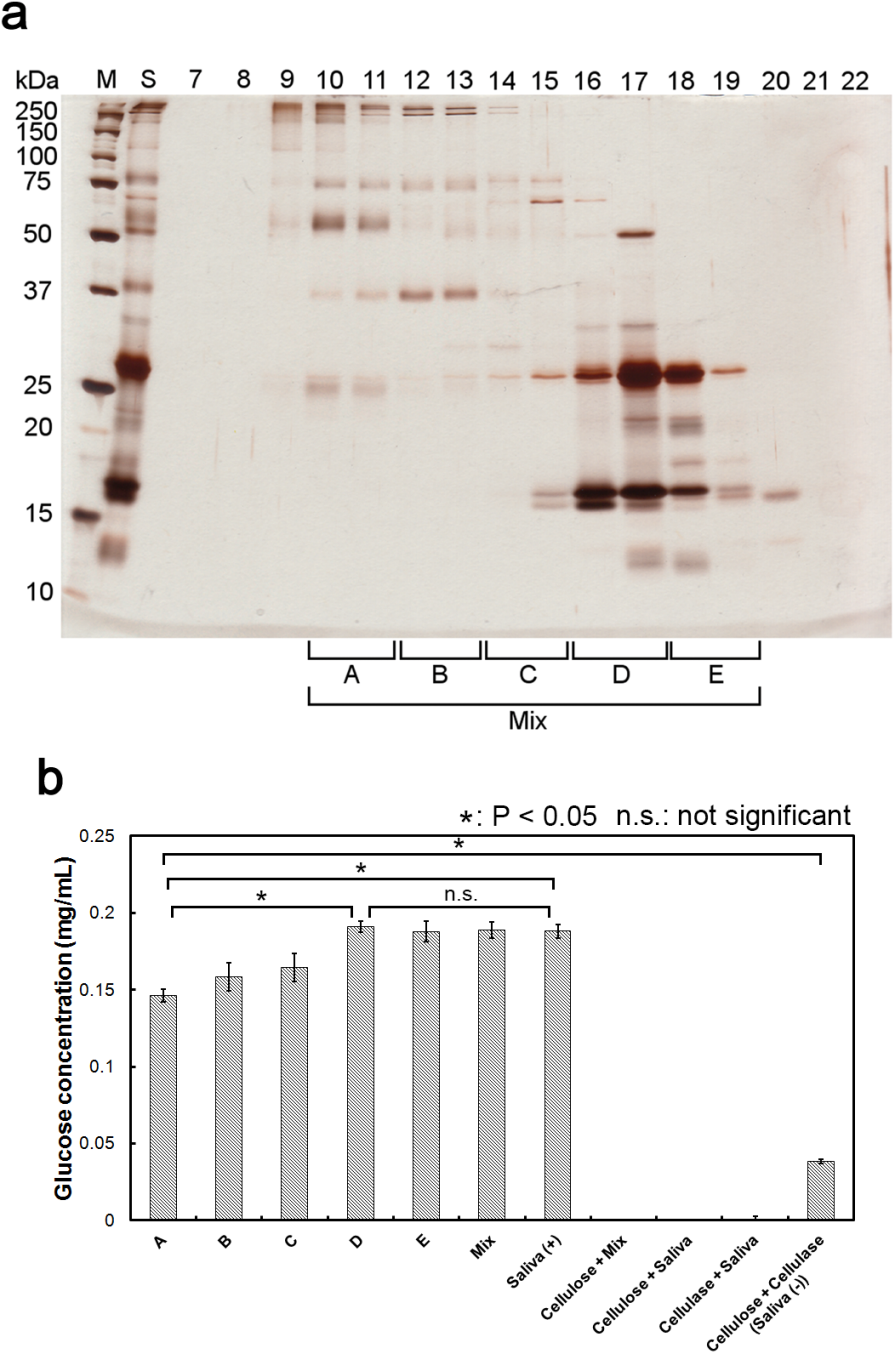
Fractionation of cattle saliva proteins. (a) SDS-PAGE analysis of fractionated cattle saliva proteins. Proteins present in the cattle saliva were fractionated by gel filtration chromatography. An aliquot of each fraction was used for SDS-PAGE analysis, following which the acrylamide gel was stained with silver stain. Collected fractions were then divided as indicated (based on molecular weight distribution of proteins) into five sample groups (called here as sample A, B, C, D and E) for further use. (b) Enhancement effect of fractionated cattle saliva. Cellulase-catalyzed cellulose degradation assay (in triplicate) was performed using one of the fractionated saliva samples (A, B, C, D, or E) or Mix sample as an additive, and the amount of reducing sugar produced in each case was measured. Error bars indicate ± standard deviations. Values labeled with asterisk are statistically different as established by Student's t-test (P < 0.05).

Finally, to identify the protein responsible for the enhancement effect, we fractionated cattle saliva protein using gel-filtration chromatography. The fractionated proteins were subsequently analyzed by SDS-PAGE (Fig 5A). As can be seen, protein bands were present in fractions 9 through 20. Based on the molecular weight distribution of the fractionated proteins, we divided these factions into five sample groups, A through E, as follows: (A) fraction numbers 10 and 11 containing proteins whose molecular weights were more than 50 kDa; (B) fraction numbers 12 and 13 containing proteins with molecular weights of approximately 37 kDa; (C) fraction numbers 14 and 15 containing proteins with molecular weights of approximately 70, 25 and 15 kDa; (D) fraction numbers 16 and 17 containing proteins with molecular weights of approximately 25 and 15 kDa; and (E) fraction numbers 18 and 19 containing proteins whose molecular weights were similar to those in (D). We also prepared a mixed sample (Mix) by combining equal volumes of fractions A through E. We then used these samples in the cellulose degradation assay and the results are shown in Fig 5B. The amount of glucose produced using samples A, B and C as additives ranged from 0.140 mg/mL to 0.170 mg/mL, whereas the amount of glucose produced using samples D, E, Mix and Cellulase + Saliva (Saliva (+)) were approximately 0.190 mg/mL. In all cases, the amount of glucose produced using any one of these samples was higher than the amount of glucose produced using mixture containing only cellulase (Cellulose + Cellulase (Saliva (-))). Because all assay mixtures contained same amount of protein, the enhancement effect appeared to decrease slightly corresponding increase of the molecular weight of the protein.

Taken together, these results suggested that the substances in cattle saliva responsible for enhancing the enzymatic activity of cellulase were probably non-enzymatic proteins.

### Mechanism of Cattle Saliva Mediated Enhancement of Cellulase Activity

**Fig 6:**
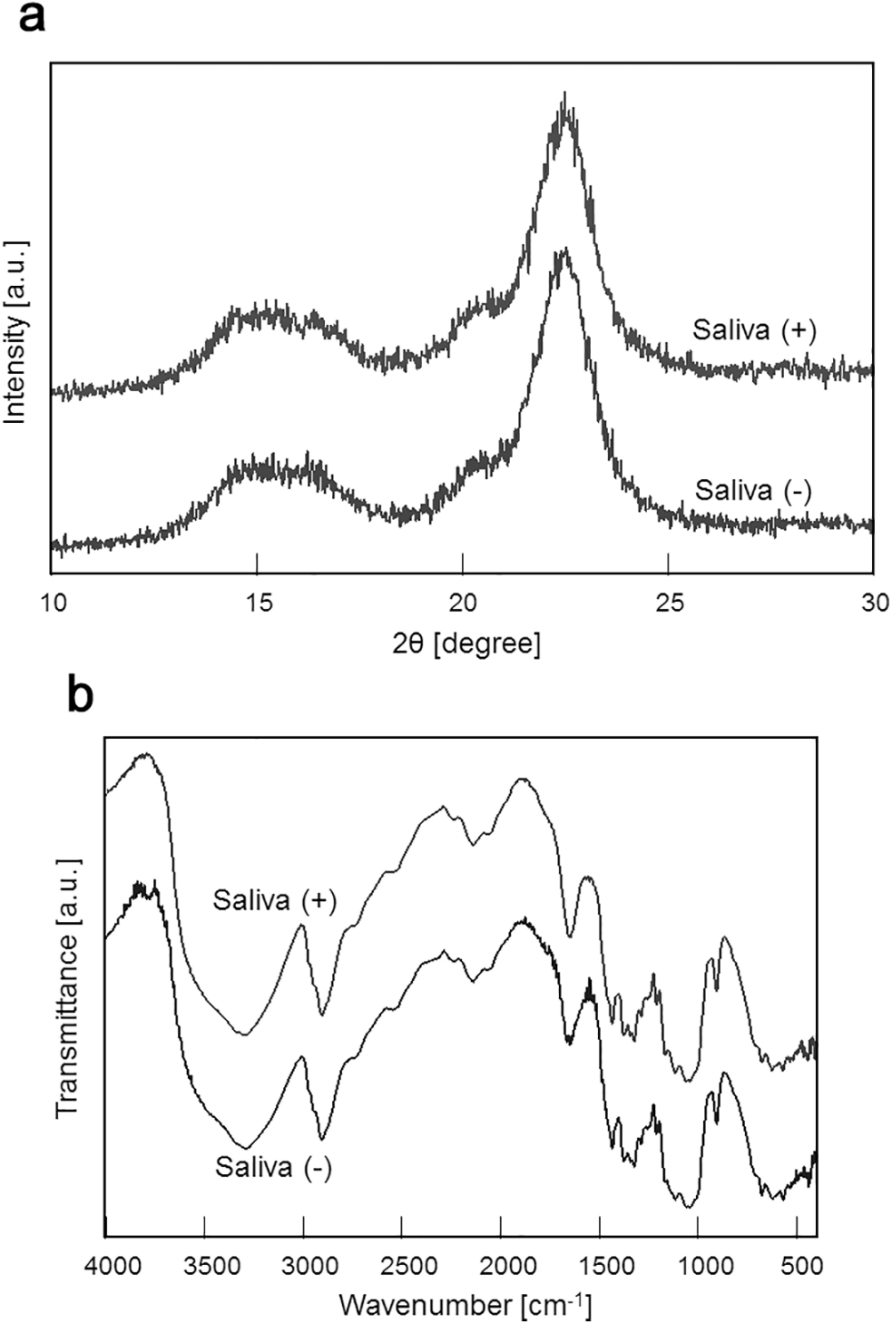
Crystal structure analysis of cellulose. The crystal structure of cellulose in the presence of cattle saliva (Saliva(+)) or in the absence of cattle saliva (Saliva(-)) was analyzed by (a) X-ray diffraction and (b) FT-IR.

Recent studies demonstrated that pretreatment of cellulose with certain types of compounds altered its crystal structure and enhanced its degradation. For example, pretreatment with liquid ammonia or amine solution transformed cellulose I structure into cellulose III structure [16, 17]. As any change in the cellulose crystal structure could be detected using the X-ray diffraction spectroscopy and FT-IR spectroscopy [18, 19], we employed these two spectroscopic methods to examine the crystal structure of cellulose in the presence (Saliva (+)) and absence (Saliva (-)) of cattle saliva in order to determine whether the addition of cattle saliva would cause any change in the crystal structure of cellulose, and the results are shown in Fig 6. X-ray diffraction analysis revealed two peaks in both mixtures: a broad peak in which 2θ was varied from 15 to 18 degree, and a sharp peak in which 2θ was approximately 23 degree (Fig 6A). No significant difference was observed between the X-ray spectra of Saliva (+) and Saliva (-) samples. FT-IR spectra of both Saliva (+) and Saliva (-) samples showed many peaks appearing at different wavenumbers (Fig 6B). However, no differences in peak intensities and spectrum shapes were observed between the Saliva (+) and Saliva (-) samples. Taken together, these results suggested that addition of cattle saliva to cellulose did not cause any macroscopic change in the crystal structure of micro-crystalline cellulose.

**Fig 7:**
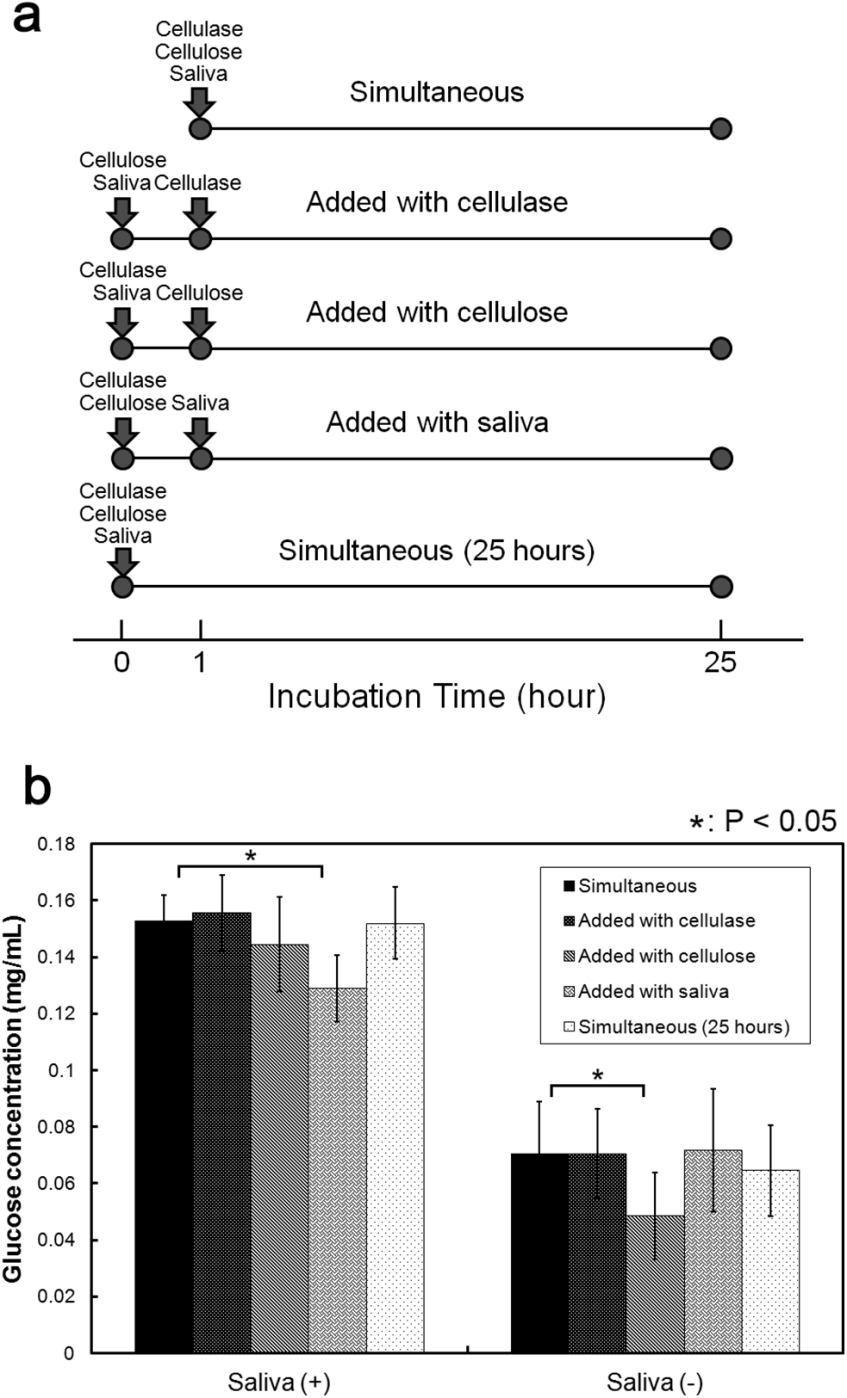
Addition order assay. (a) Schematic representation of the experimental design. (b) Effect on the production of reducing sugar. The amount of reducing sugar produced at each addition order experimental condition, shown schematically in (a), was measured. Simultaneous: A mixture in which cellulose, cellulase and cattle saliva were added simultaneously. Added with cellulase: Cellulase was added to a mixture containing cellulose and cattle saliva. Added with cellulose: Cellulose was added to a mixture containing cellulase and cattle saliva. Added with saliva: Cattle saliva was added to a mixture containing cellulose and cellulase. Simultaneous (25 hours): A mixture in which cellulose, cellulase and cattle saliva were added simultaneously and incubated for 25 h. Error bars indicate ± standard deviations (n = 9). Values labeled with asterisk are statistically different as established by Student's t-test (P < 0.05).

**Fig 8:**
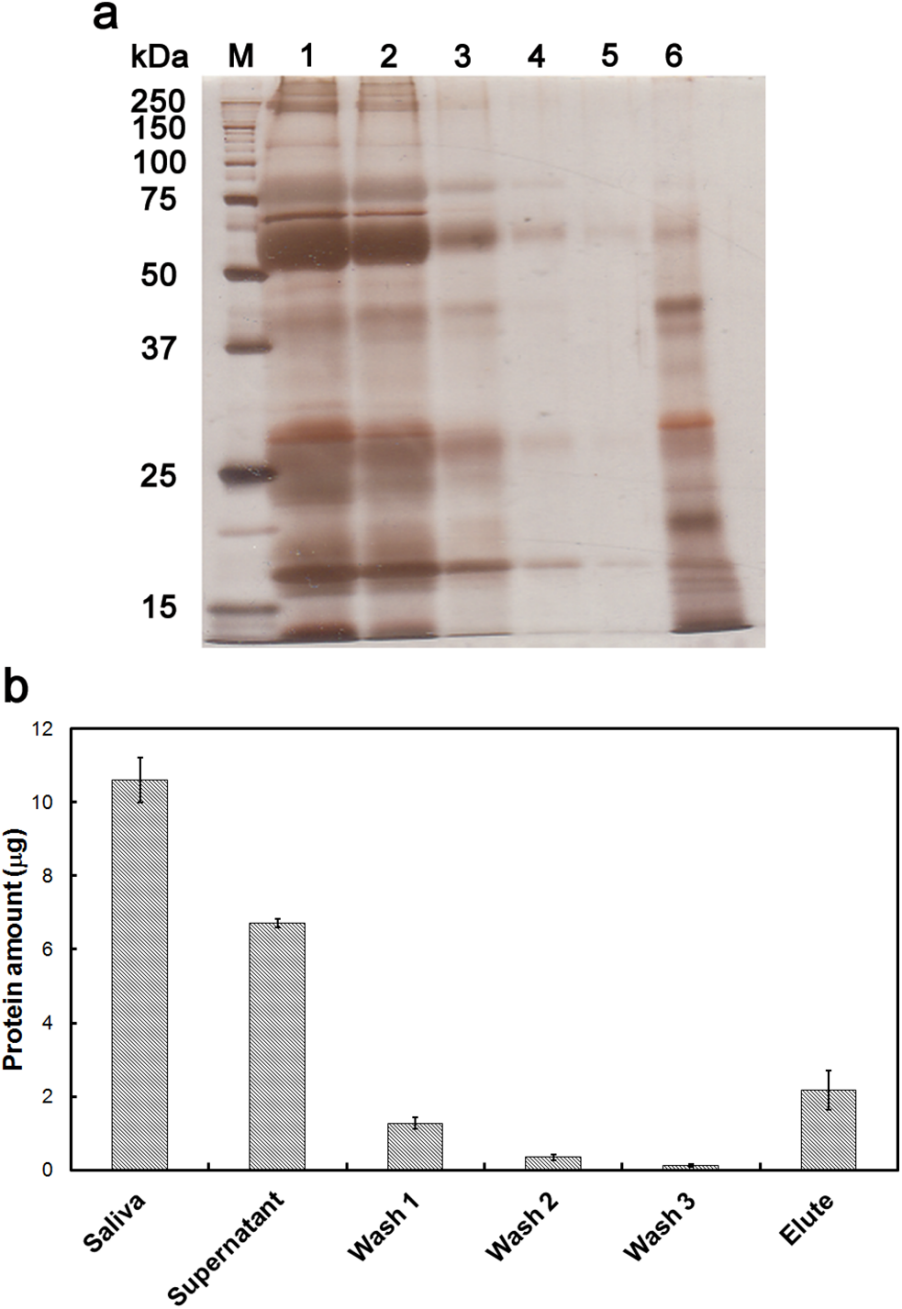
Adsorption analysis of cattle saliva proteins. Adsorption of cattle saliva proteins to cellulose was analyzed using (a) SDS-PAGE and (b) Bradford protein assay. (a) SDS-PAGE analysis. Lane 1: Cattle saliva solution (80%). Lane 2: Supernatant, supernatant after the mixture was incubated at 50°C for an hour. Lane 3: Wash 1, supernatant of Wash buffer 1. Lane 4: Wash 2, supernatant of Wash buffer 2. Lane 5: Wash 3, supernatant of Wash buffer 3. Lane 6: Elute, eluted fraction after the cellulose pellet was mixed with 0.5% SDS and incubated at 96°C for 1 h. (b) Amount of protein in each sample used for SDS-PAGE analysis was quantified by Bradford protein assay. Error bars indicated ± deviations (n = 3).

In order to delineate the mechanism of cattle saliva mediated enhancement of cellulase activity, we thought that it was necessary to determine whether cattle saliva interacts with cellulase, cellulose or both. Thus, we carried out an addition order experiment performed according to the strategy shown in Fig 7A. Results obtained from this addition order experiment were shown in Fig 7B. First, in the absence of cattle saliva (Saliva (-)), the amount of glucose produced under the experimental conditions ‘Simultaneous’, ‘Added with cellulase’, ‘Added with saliva’ and ‘Simultaneous (25 h)’ was approximately 0.070 mg/mL, whereas the amount of glucose produced under the ‘Added with cellulose’ condition was 0.049 mg/mL. The decrease in cellulase activity under the ‘Added with cellulose’ condition was probably because cellulase was diluted and it became partly denatured as a result of incubation at 50°C for 1 h in the absence of any cellulose molecule to interact with. Second, in the presence of cattle saliva (Saliva (+)), the amount of glucose produced under experimental conditions ‘Simultaneous’, ‘Added with cellulase’, ‘Added with cellulose’, and ‘Simultaneous (25 h)’ was 0.150 mg/mL, whereas the amount of glucose produced under the ‘Added with saliva’ condition was 0.129 mg/mL, indicating that the enhancement effect of cattle saliva was slightly decreased when the cattle saliva was added at a later stage. Thus, the production of reducing sugar, to some extent, was dependent on the addition order by which the components were mixed. The observed result also suggested that cattle saliva needed to interact with cellulose before it was degraded by cellulase. In order to confirm that cattle saliva interacts with cellulose, we used SDS-PAGE analysis and Bradford protein assay to determine whether cattle saliva proteins would get adsorbed to cellulose. As shown in Fig 8, proteins were detected in the Supernatant, Wash 1, Wash 2 and Wash 3 fractions, but the amount of proteins in these fractions decreased with the number of wash; however, more proteins were eluted off the cellulose after it was mixed with SDS and the mixture was incubated at 96°C for 1 h. Thus, several cattle saliva proteins were adsorbed to cellulose. Taken together, these results suggested that the enhancement effect was caused by the adsorbed non-enzymatic proteins in cattle saliva.

**Fig 9:**
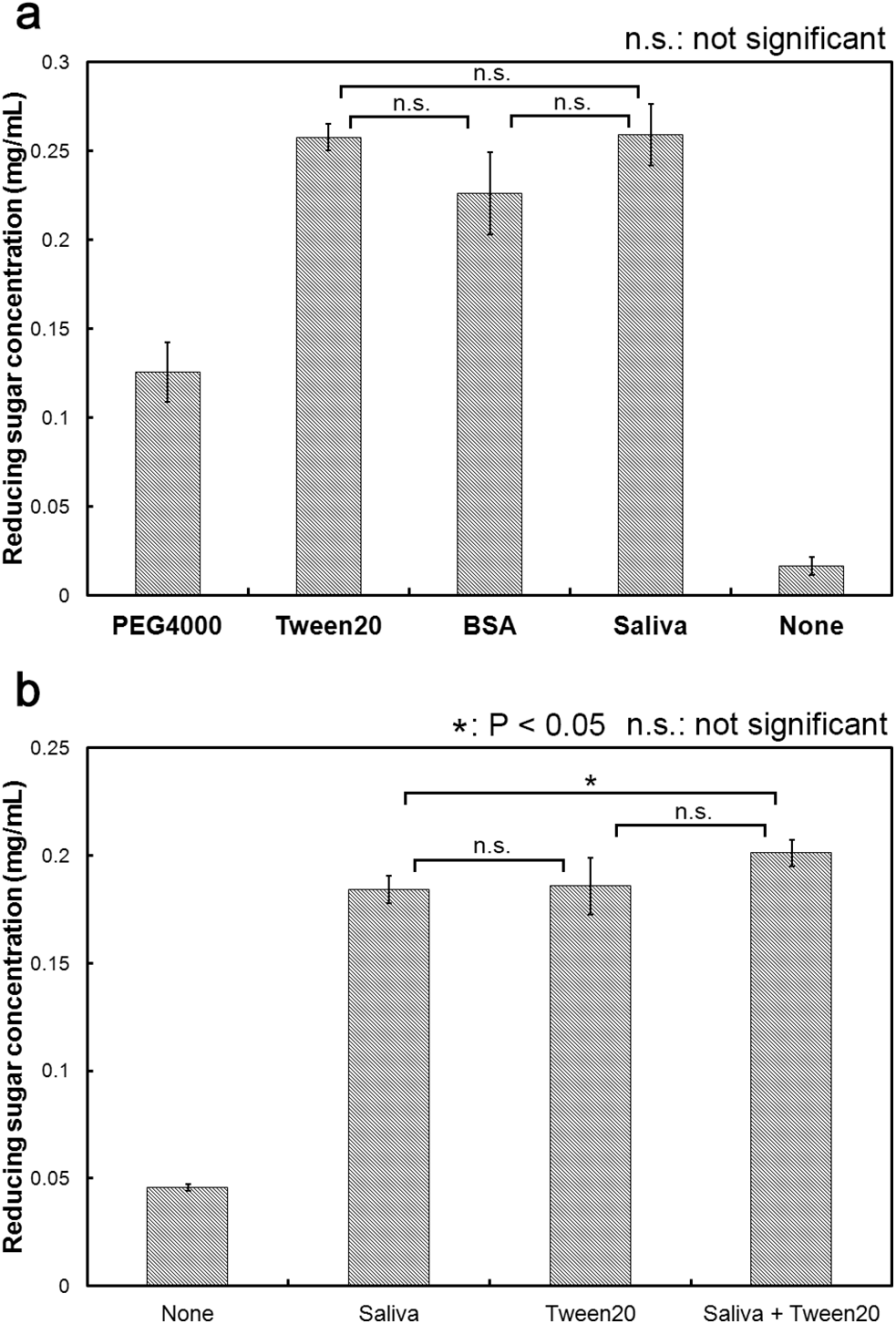
Comparison and competition between cattle saliva and canonical additives. (a) Comparison between the enhancing effects of cattle saliva and canonical additives. The amount of reducing glucose produced was measured using optimal concentration one of the following additives: PEG 4000 (50 mg/mL), Tween 20 (2.5 mg/mL) BSA (0.01 mg/mL), cattle saliva (7.75%) or none. (b) Competition between cattle saliva and Tween 20. Concentrations of cattle saliva and Tween 20 required for saturating the cellulase catalyzed production of reducing sugar were 7.75% and 2.5 mg/mL, respectively. All experiments were performed in triplicate and error bars indicate ± standard deviations. Values labeled with asterisk are statistically different as established by Student's t-test (P < 0.05).

Several previous studies reported that many different types of additives enhanced the degradation of cellulose. For example, cellulase-catalyzed degradation of cellulose was enhanced by the addition of PEG 4000 [8, 20], Tween 20 [10, 21] or BSA [11, 22]. Therefore, we next confirmed that these three additives indeed enhanced cellulose degradation. First, using various concentrations of PEG 4000 (2.5, 12.5, 25, and 50 mg/mL), we measured the amount of reducing sugar produced, the maximum concentration of which was found to be 0.126 mg/mL at 50 mg/mL of PEG 4000. Second, using various concentrations of Tween 20 (2.5, 12.5, 25, and 50 mg/mL), we measured the amount of reducing sugar produced, the maximum concentration of which was found to be 0.258 mg/mL at 2.5 mg/mL of Tween 20. Third, using various concentrations of BSA (0.005, 0.01, 0.05, 0.1,and 0.5 mg/mL), we measured the amount of sugar produced, the maximum concentration of which was found to be 0.226 mg/mL at 0.01 mg/mL of BSA. These maximum sugar concentration values were plotted again in Fig 9A. As can be seen, the production of reducing sugar was increased 7.5-fold by the addition of PEG 4000, and more than 13.5-fold by the addition of Tween 20, BSA or cattle saliva. These results confirmed the earlier reported observation that the enzymatic activity of cellulase was enhanced by certain types of polymers, proteins and surfactants. In order to further understand the underlying mechanism of the enhancement effect of cattle saliva, we next examined whether cattle saliva could compete with Tween 20. Results of the competition assay were shown in Fig 9B. Since the amount of reducing sugar produced became saturated when we used more than 4% of cattle saliva (Fig 1D) or 2.5 mg/mL of Tween 20 (Fig 9A) as additives, we therefore used 7.75% cattle saliva and 2.5 mg/mL Tween 20 as additives for the competition assay. As shown in Fig 9B, the amount of reducing sugar produced with saliva, Tween 20 and saliva + Tween 20 were 0.184 mg/mL, 0.186 mg/mL and 0.201 mg/mL, respectively. The amount of reducing sugar produced with saliva + Tween 20 was not significantly different compared to that with Tween 20, but was slightly different from that with saliva. The produced sugar rate using saliva was 92% based on using saliva + Tween 20, although saliva + Tween 20 mixture additionally contained Tween 20 compared with saliva mixture. This slight difference was not important to discussing the result of competition assay. Thus, cattle saliva was able to compete with Tween 20 for exerting its enhancing effect. This result suggested that the enhancement effect of cattle saliva possibly mediated via a mechanism that is similar to that of Tween 20. Proteins are composed of various types of hydrophobic and hydrophilic amino acid residues. Thus, just as in surfactants, proteins also have hydrophobic and hydrophilic parts and hence they may behave like surfactants.

## Conclusion

Cattle saliva enhanced cellulase-catalyzed degradation of cellulose (Fig 1A). The enhancement effect saturated at high cellulase as well as high cattle saliva concentrations (Fig 1B and 1D), and the enhancement was more apparent at shorter incubation times (less than 24 hours) (Fig 1C). Substances responsible for this enhancement effect were non-enzymatic proteins (Figs 3–5). We also found that cattle saliva did not induce any structural change at the macroscopic level in the crystal structure of micro-crystalline cellulose (Fig 6), and the enhancement effect was caused by the adsorbed non-enzymatic proteins, which might have behaved like surfactants (Figs 7–9). A previous study have shown that after a repeated number of reversible binding to cellulose, cellulase eventually gets bound to cellulose irreversibly and, as a result, it becomes inactive [23]. This adsorption of cellulase prevented further hydrolysis of cellulose [24]. Based on our results, we propose the following mechanism by which cattle saliva enhances the cellulase catalyzed hydrolysis of cellulose. Surfactant-like effect of adsorbed cattle saliva proteins enhances the surface activity at the cellulose-liquid interface and thereby keeps the activity of celluase stable during the cellulose degradation process. As a result, cellulase can carry out the processes of adsorption to cellulose, degradation of cellulose and desorption from cellulose repeatedly and more smoothly. At longer reaction times, however, many cellulase molecules become inactive and remain irreversibly bound to cellulose. Thus, the cellulose degradation process cannot accelerate in spite of the presence of absorbed cattle saliva proteins. Actually, the following observations support this proposed mechanism: the enhancement effect was saturated at high concentration of cellulase (Fig 1B) and the effect was more pronounced at shorter incubation times (Fig 1C). In the addition order experiment, the activity of cellulase in the ‘Added with saliva’ reaction mixture did not accelerate at the earlier stage of the reaction because of the absence of cattle saliva in the mixture. As a result, the amount of glucose produced in the ‘Added with saliva’ reaction mixture was less compared to the amount of glucose produced in the ‘Simultaneous’ or ‘Added with cellulase’ reaction mixture. We believe that cellulose degradation was promoted by this mechanism, which is one of the mechanisms that could explain the enhancement effect of cattle saliva. A number of previous studies have proposed several other mechanisms to explain the enhancement effect of surfactants. For example, Kaar and Holtzapples [12] suggested that Tween prevented the thermal deactivation of cellulase. Kim et al. [13], on the other hand, suggested that surfactants compete with the cellulase for the air-liquid interface and thereby protect cellulase from deactivation. The enhancement effect of cattle saliva is probably caused by a combined effect of these mechanisms. A similar enhancement effect of cattle saliva also occurred for the real biomass substrate (Fig 2). However, the enhancement rate for the real biomass substrate (1.2-fold) was lower than for pure cellulose (2.9-fold) (Figs 1A and 2A). The real biomass substrate contains various kinds of organic substances and minerals, including protein (approximately 10%) [14]. The enhancement effect caused by the protein in the real biomass may compete with the enhancement effect caused by the non-enzymatic protein in cattle saliva.

Many additives have been shown to enhance the degradation of cellulose [10]. The biggest advantages of using cattle saliva as an additive, compared to using other canonical additives, are that the cattle saliva is easily available at a low cost and in large amounts, as there are many cows all over the world. Furthermore, there is no need to purify cattle saliva for its use. On the other hand, canonical additives, such as surfactants, have to be synthesized. This prevents surfactants from being available in large amounts at a low cost. Thus, cattle saliva could serve as a promising additive for efficient hydrolysis of cellulose on an industrial scale.

## References

[1] Duff S, Murray W (1996) Bioconversion of forest products industry waste cellulosics to fuel ethanol: A review. Bioresour Technol 55:1–33.

[2] Lynd LC, Cushman JH, Nichols RJ, Wyman CE (1991) Fuel ethanol from cellulosic biomass. Science 251:1318–1323. 17816186 10.1126/science.251.4999.1318

[3] Fan LT, Lee YH, Beardmore DH (1980) Mechanism of the enzymatic hydrolysis of cellulose: Effects of major structural features of cellulose on enzymatic hydrolysis. Biotechnol Bioeng 22:177–199.

[4] Castanon M, Wilke CR (1981) Effects of the surfactant Tween-80 on enzymatic-hydrolysis of newspaper. Biotechnol Bioeng 23:1365–1372.

[5] Ooshima H, Sakata M, Harano Y (1986) Enhancement of enzymatic hydrolysis of cellulose by surfactant. Biotechnol Bioeng 28:1727–1734. 18555287 10.1002/bit.260281117

[6] Kaya F, Heitmann JA, Joyce TW (1995) Influence of surfactants on the enzymatic-hydrolysis of xylan and cellulose. Tappi J 78:150–157.

[7] Kristensen JB, Börjesson J, Bruun MH, Tjerneld F, Jørgensen H (2007) Use of surface active additives in enzymatic hydrolysis of wheat straw lignocelluloses. Enzyme Microb Technol 40:888–895.

[8] Zhang Y, Xu X, Zhang Y, Li J (2011) Effect of adding surfactant for transforming lignocellulose into fermentable sugars during biocatalysing. Biotechnol Bioproc E 16:930–936.

[9] Helle SS, Duff SJB, Cooper DG (1993) Effect of surfactants on cellulose hydrolysis. Biotechnol Bioeng 42:611–617. 18613083 10.1002/bit.260420509

[10] Eriksson T, Börjesson J, Tjerneld F (2002) Mechanism of surfactant effect in enzymatic hydrolysis of lignocellulose. Enzyme Microb Technol 31:353–364.

[11] Yang B, Wyman CE (2006) BSA Treatment to Enhance Enzymatic Hydrolysis of Cellulose in Lignin Containing Substrates. Biotechnol Bioeng 94:611–617. 16673419 10.1002/bit.20750

[12] Kaar WE, Holtzapple MT (1998) Benefits from Tween during enzymic hydrolysis of corn stover. Biotechnol Bioeng 59:419–427. 10099355 10.1002/(sici)1097-0290(19980820)59:4<419::aid-bit4>3.0.co;2-j

[13] Kim MH, Lee SB, Ryu DDY, Reese ET (1982) Surface deactivation of cellulase and its prevention. Enzyme Microb Technol 4:99–103.

[14] Chiquette J, Flipot PM, Vinet CM (1992) Effect of ammoniation and urea addition on chemical composition and digestibility of mature timothy hay, and rumen fluid characteristics of growing steers. Can J Anim Sci 72:299–308.

[15] Miwa I, Okuda J, Maeua K, Okuda G (1972) Mutarotase effect on colorimetric determination of blood glucose with β-D-glucose oxidase. Clin Chim Acta 37:538–540. 5063213 10.1016/0009-8981(72)90483-4

[16] Barry AJ, Peterson FC, King AJ (1936) x-Ray Studies of Reactions of Cellulose in Non-Aqueous Systems. I. Interaction of Cellulose and Liquid Ammonia. J Am Chem Soc 58:333–337.

[17] Davis WE, Barry AJ, Peterson FC (1943) X-Ray Studies of Reactions of Cellulose in Non-Aqueous Systems. II. Interaction of Cellulose and Primary Amines. J Am Chem Soc 65:1294–1299.

[18] Hattori K, Abe E, Yoshida T, Cuculo JA (2004) New Solvents for Cellulose. II.Ethylenediamine/Thiocyanate Salt System. Polym J 36:123–130.

[19] Yan L, Gao Z (2008) Dissolving of cellulose in PEG/NaOH aqueous solution. Cellulose 15:789–796.

[20] Börjesson J, Engqvist M, Sipos B, Tjerneld F (2007) Effect of poly(ethylene glycol) on enzymatic hydrolysis and adsorption of cellulase enzymes to pretreated lignocellulose. Enzyme Microb Technol 41:186–195.

[21] Park JW, Takahata Y, Kajiuchi T, Akehata T (1992) Effects of nonionic surfactant on enzymatic hydrolysis of used newspaper. Biotechnol Bioeng 39:117–120. 18600894 10.1002/bit.260390117

[22] Wang H, Mochidzuki K, Kobayashi S, Hiraide H, Wang X, Cui Z (2013) Effect of bovine serum albumin (BSA) on enzymatic cellulose hydrolysis. Appl Biochem Biotechnol 170:541–551. doi: 10.1007/s12010-013-0208-0 23553108 10.1007/s12010-013-0208-0

[23] Klyosov AA, Rabinowitch ML (1980) Enzymatic conversion of cellulose to glucose: present state of the art and potential. Enzyme Eng: Future Directions 83–165.

[24] Howell JA, Mangat M (1978) Enzyme deactivation during cellulose hydrolysis. Biotechnol Bioeng 20:847–863.

